# Down-regulation of p21-activated serine/threonine kinase 1 is involved in loss of mesencephalic dopamine neurons

**DOI:** 10.1186/s13041-016-0230-6

**Published:** 2016-04-27

**Authors:** Hwanhee Kim, Jun-Young Oh, Sun-Lim Choi, Yeon-Ju Nam, Anna Jo, Ara Kwon, Eun-Young Shin, Eung-Gook Kim, Hyong Kyu Kim

**Affiliations:** Department of Medicine and Microbiology, College of Medicine, Signaling Disorder Research Center, Chungbuk National University, Cheongju, 28644 The Republic of Korea; Department of Medicine and Biochemistry, College of Medicine, Signaling Disorder Research Center, Chungbuk National University, Cheongju, 28644 The Republic of Korea; Department of Medicine and Microbiology, College of Medicine, Chungbuk National University, Chungdae-ro 1, Seowon-gu, Cheongju, 28644 The Republic of Korea

**Keywords:** PAK1, Bcl-2, Apoptosis, Neurodegeneration, Parkinson’s disease

## Abstract

**Background:**

Although the roles of p21-activated serine/threonine kinase 1 (PAK1) have been reported in some neurodegenerative diseases, details regarding neurodegeneration are still limited. Hence, we tried to determine the role of PAK1 and molecular mechanisms of neuronal death involved in neurodegeneration.

**Results:**

Expression of a dominant-negative form of PAK1 (PAK1^H83,86L, K229R^, PAK1-DN) decreased the cell viability and increased cell death induced by oxidative stress. Indeed, oxidative stress decreased the phosphorylation of PAK1 in neuroblastoma cells, cultured dopamine (DA) neurons, or rat midbrains. PAK1-DN reduced the level of Bcl-2 protein, through an ubiquitin/proteasome-dependent mechanism. The level of Bcl-2 may be regulated by PAK1-ERK signaling and/or PAK1, directly. Conversely, expression of an active form of PAK1 (PAK1^T423E^, PAK1-CA) could recover both loss of DA neurons in the substantia nigra (SN) and behavioral defects in a 6-OHDA-induced hemiparkinsonian rat model.

**Conclusions:**

Our data suggest that the oxidative stress-induced down-regulation of PAK1 activity could be involved in the loss of mesencephalic DA neurons through modulation of neuronal death, suggesting a novel role of PAK1 as a molecular determinant and mechanisms in the pathogenesis of Parkinson’s disease.

**Electronic supplementary material:**

The online version of this article (doi:10.1186/s13041-016-0230-6) contains supplementary material, which is available to authorized users.

## Background

The p21-activated serine/threonine kinase 1 (PAK1) has a role in a variety of cellular functions like cell motility, cell cycle, survival, and even death. In cancer cells, PAK has functional roles in growth, invasion, and metastasis [1–5]. Accordingly, the activity of PAK1 is up-regulated through protein overexpression or gene amplification in cancer tissues [4, 5]. Although the roles of PAK1 have been widely studied in a variety of cancers and cancer cells, other roles of PAK1 still remain to be elucidated [6].

Tumor formation and progression requires changes in cellular events such as the expression of oncogenes, activation of anti-apoptotic signaling pathways, and simultaneous inactivation of pro-apoptotic signals as well as of tumor suppressors. Apoptosis (type I cell death), also known as programmed cell death, is a finely regulated mechanism initiated by signaling in response to a variety of stresses cells. Thus, it has important roles in development and tissue homeostasis in multicellular organisms. The susceptibility to apoptotic cell death is dependent on the relative level of anti-apoptotic proteins of Bcl-2 family members (Bcl-2, Bcl-x_L_, Mcl-1) to that of pro-apoptotic proteins of the family (Bad, Bax, Bak, Bok) or the interaction of each protein. Anti-apoptotic protein Bcl-2, which acts as a proto-oncogene, is a member of protective complexes that help to maintain the integrity of mitochondrial membrane. Pro-apoptotic proteins, such as Bad and Bax, can bind to Bcl-2. This binding inhibits the function of Bcl-2 and induces the release of pro-apoptotic factors by neutralizing the effects of pro-apoptotic proteins, and cell death eventually [7–9]. PAK1 affects the activity of pro-apoptotic signals in a Raf-1 dependent or independent manner. Raf-1 activated by PAK1 is translocalized to the mitochondria, and then it phosphorylates Bad, which is a pro-apoptotic protein [10]. Alternatively, PAK1 directly phosphorylates Bad on Ser112 and Ser136, inducing inhibition of the interaction with Bcl-2 or Bcl-x_L_ and subsequently resulting in cell survival [11].

In the nervous system, dysregulation of PAK signaling is implicated in neurodegenerative diseases, such as Alzheimer’s disease (AD) and Huntington’s disease (HD), and mental retardation [12]. Down-regulation of PAK1 and PAK3, brain specific forms, has been reported in AD brain [13]. Also, PAK1 overexpression enhanced the toxicity in a HD cellular model [14]. Rac-PAK signaling was found to be defective in the mouse model of Fragile X syndrome, *Fmr1*-KO mouse, suggesting that PAK1 might also be involved in the pathogenesis of mental retardation [15]. In addition, knockout of PAK1 induced defects in brain development [16]. However, up to now, molecular details involved in Parkinson’s disease (PD) are limited.

It has been widely thought that cancer or neurodegenerative disease may originate from opposite fate of cells, to death or persistent growth, respectively. As a matter of fact, some neurodegenerative diseases, such as PD, have an evident inverse co-morbidity with many cancers [17]. A certain protein, known as a genetic determinant, may decide directions of the disease to either cancers or neurodegenerative diseases according to the cellular condition in which the determinant is activated or inactivated, or the cell type, whether or not the cells have the ability to proliferate in response to the same external stimuli that can induce DNA damage or cell cycle activation [18]. Even importance of genetic determinants or molecular determinants in the pathogenesis of such diseases, not much research has been performed.

The contradictory roles of PAK1 in cancers and AD and in cell survival and cell death suggest the possibility of PAK as a new molecular determinant whose activity could determine cell fate. Hence, we examined the role of PAK1 in neuronal death. Here, we report novel molecular mechanisms involved in the death of mesencephalic DA neurons mediated by PAK1 and suggest a new molecular target for a therapeutic agent in PD.

## Results

### Down-regulation of PAK1 activity increases apoptotic cell death

**Fig. 1:**
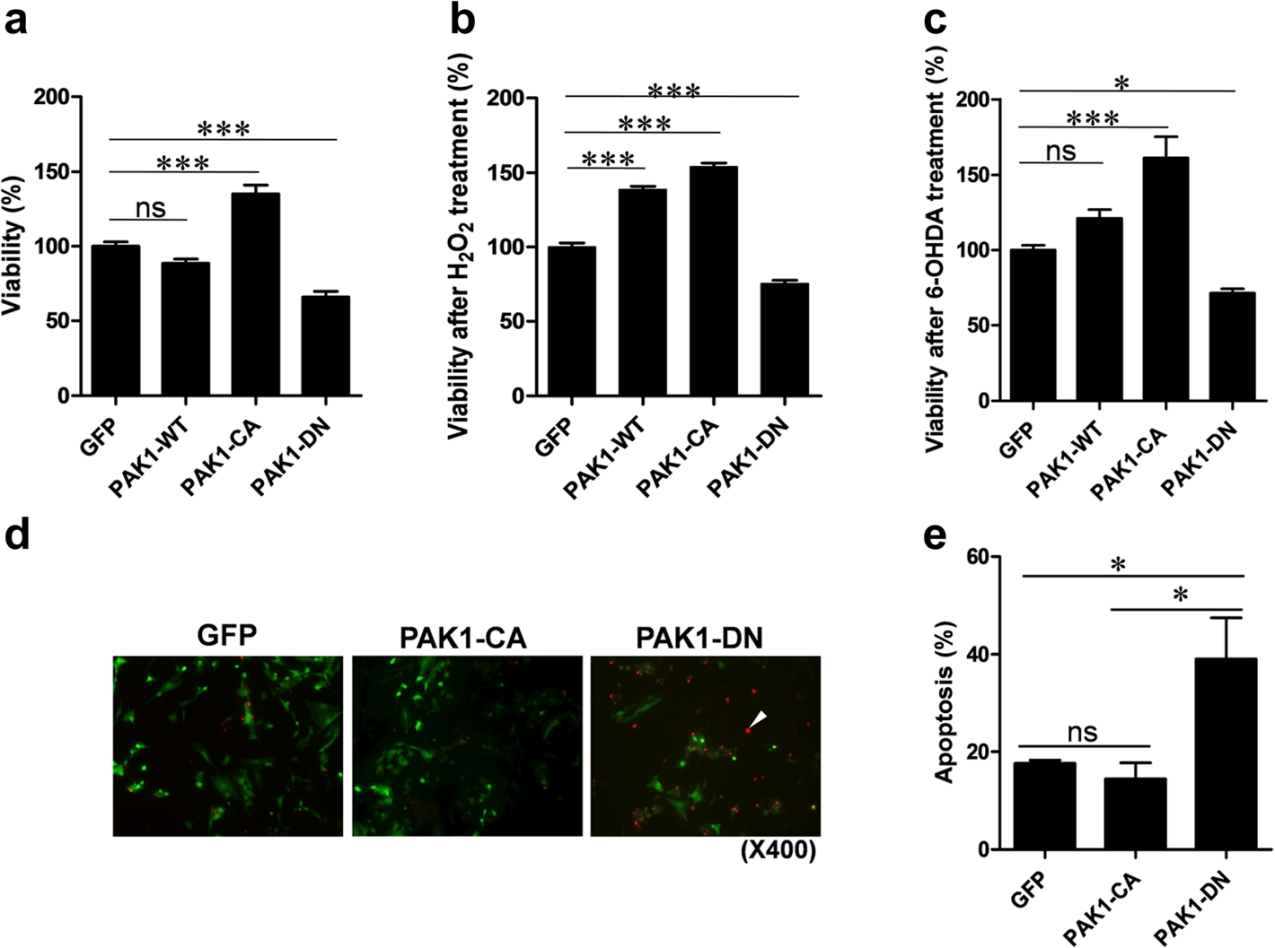
PAK1-DN expression reduces viability. **a** PAK1-DN expression reduced viability of SH-SY5Y cells but expression of PAK1-CA mutant increased viability of Sh-SY5Y cells (GFP: 100.0 ± 2.99 %, *n =* 4; WT: 88.6 ± 2.84 %, *n =* 4; CA: 135.1 ± 6.03 %, *n =* 4; DN: 65.94 ± 3.80 %, *n =* 4; One-way ANOVA, *F*_3,15_ = 49.14, ^****^ *P* < 0.0001, Newman-Keuls multiple comparison test: ^***^ *P* < 0.001, ns: not significant). **b** PAK1-DN expression reduced viability of SH-SY5Y cells after oxidative stress induced by treatment with hydrogen peroxide (GFP: 100.0 ± 2.68 %, *n =* 12; WT: 138.7 ± 2.13 %, *n =* 12; CA: 154.1 ± 2.52 %, *n =* 12; DN: 75.41 ± 2.18 %, *n =* 12; One-way ANOVA, *F*_3,47_ = 225.6, ^****^ *P* < 0.0001, Newman-Keuls multiple comparison test: ^***^ *P* < 0.001). **c** PAK1-DN expression reduced viability of SH-SY5Y cells after oxidative stress induced by treatment with 6-hydroxydopamine (GFP: 100.0 ± 3.12 %, *n =* 4; WT: 121.2 ± 5.73 %, *n =* 4; CA: 161.3 ± 14.11 %, *n =* 4; DN: 71.35 ± 2.92 %, *n =* 12; One-way ANOVA, *F*_3,15_ = 22.95, ^****^ *P* < 0.0001, Newman-Keuls multiple comparison test: ^*^ *P* < 0.05, ^***^ *P* < 0.001, ns: not significant). **d** PAK1-DN expression significantly increased apoptotic cell death. At 72 h after plating of SH-SY5Y cells expressing PAK1 mutants, the cells were subjected to TUNEL staining. **e** Bar graphic representation of TUNEL staining results (GFP: 17.6 ± 30.63 %, *n =* 3; CA: 14.4 ± 3.31 %, *n =* 3; DN: 39.08 ± 8.46 %, *n =* 3; One-way ANOVA, *F*_2,8_ = 6.50, *P* = 0.0315, Newman-Keuls multiple comparison test: ^*^ *P* < 0.05)

In order to determine the role of PAK1 in cell proliferation and viability, we constructed the cell lines stably expressing wild-type PAK1 (WT), constitutively active PAK1 mutant (PAK1-CA, PAK1^T423E^), dominant-negative PAK1 mutant (PAK1-DN, PAK1^H83,86L, K299R^) [19, 20], or GFP using a neuroblastoma cell line, SH-SY5Y cell line, lentiviral vector, and fluorescence-activated cell sorting. The SH-SY5Y cells strongly expressed endogenous PAK1 (Additional 1: Figure S1). First, to examine the viability of each cell line, the same number of cells (1X10^5^ cells) were plated and incubated for 72 h and the amount of viable cells was measured. As expected, the expression of PAK1-CA increased the cell viability to 35 %, but the expression of PAK1-DN decreased the cell viability to 35 % compared to that of GFP-expressing cells (Fig. 1a). In addition, these effects were recapitulated when cell death was induced by oxidative stress, such as treatment with hydrogen peroxide (H_2_O_2_) or 6-hydroxydopamine (6-OHDA) (Fig. 1b, c). These data indicate that down-regulation of PAK1 activity enhances both cell death and vulnerability to oxidative stress. Subsequently, we examined the cell death mechanism which might be involved in the decrease of cell viability induced by PAK1-DN expression. At 72 h after plating, SH-SY5Y cells expressing PAK1 mutants were subjected to TUNEL staining. Apoptotic cell death in PAK1-DN-expressing cells was increased by approximately 118 %, compared to that in GFP-expressing control cells. On the contrary, apoptotic cell death in PAK1-CA expressing cells was decreased compared to that in the control cells, but the difference was not significant. These results indicate that PAK1 activity could regulate apoptotic cell death in neuroblastoma cells.

### PAK1 is inactivated by oxidative stress

**Fig. 2:**
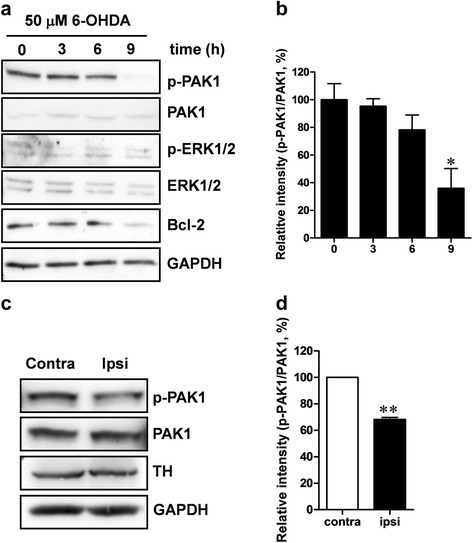
Oxidative stress induced by 6-OHDA treatment reduces phosphorylated PAK1. **a** SH-SY5Y cells were treated with 50 μM of 6-OHDA and harvested at 0, 3, 6, and 9 h after treatment. The lysates were used to measure phosphorylated PAK1 and total PAK1 levels by Western blot analysis. **b** Bar graphic representation of Western blot analysis [0 h: 100.0 ± 11.61 %, *n =* 3; 3 h: 95.4 ± 5.35 %, *n =* 3; 6 h: 78.2 ± 10.76, *n =* 3; 9 h: 36.0 ± 14.19 %, *n =* 3, One-way ANOVA, *F*_3,11_ = 7.070, *P* = 0.0122, Newman-Keuls multiple comparison test: ^*^ *P* < 0.05]. **c** A 4 μl dose of 6-OHDA (5 μg/μl) was infused into the right striatum at rate of 0.5 μl/min. After 7 days, the midbrain regions including the substantia nigra of the ipsilateral side and contralateral side were isolated and 30 μg of each protein was used for Western blot analysis. **d** Bar graphic representation of Western blotting analysis (Contra: 100.0 %, *n =* 3; Ipsi: 68.2 ± 1.56 %, *n =* 3, Paired *t*-tests, ** *P* < 0.001)

Oxidative stress induces cell death, either necrosis (type 2 cell death) or apoptosis (type 1 cell death), in a variety of cells. In our experiments, PAK1-DN expression increased cell death induced by treatment with H_2_O_2_ or 6-hydroxydopamine (6-OHDA), but PAK1-CA expression decreased cell death (Fig. 1b, c). Hence, we examined whether oxidative stress can regulate the activity of PAK1. In order to address this question, we examined the relative level of phosphorylated PAK1 (p-PAK1) to that of total PAK1 induced by 50 μM 6-OHDA treatment in SH-SY5Y cells at several time points. The 9 h-treatment with 6-OHDA significantly decreased the value of p-PAK1/PAK1 and this reduction was correlated with the reduction in the of Bcl-2 level (Fig. 2a, b). In addition, the reduction in the Bcl-2 level was also dependent on post-transcriptional mechanisms (Additional file 2: Figure S2). The inactivation of PAK1 by oxidative stress, such as 6-OHDA treatment, was further examined in an animal model. At one week after 20 μg of 6-OHDA was infused into the striatum of rats, the midbrain regions were isolated and subjected to Western blot analysis for p-PAK1 and PAK1. The 6-OHDA treatment significantly decreased the value of p-PAK1/PAK1 in the midbrains (Fig. 2c, d). Next, in order to exclude the effects of PAK1 in glial cells, we examined the level of p-PAK1 in response to 6-OHDA treatment in cultured mesencephalic DA neurons. Consistent with our previous data, the 6-OHDA treatment decreased the p-PAK1 level in TH-positive neurons, but it did not decrease the PAK1 level (Additional file 3: Figure S3). These results show that phosphorylation of PAK1 is decreased by oxidative stress.

### PAK1 activity could be recovered by PP2B inhibition

**Fig. 3:**
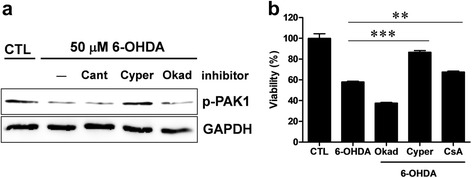
PP2B inhibitor reverses the down-regulation of PAK1 activity induced by oxidative stress. **a** SH-SY5Y cells were treated with 50 μM 6-OHDA along with 200 nM Cantharidic acid, 0.2 nM Cypermethrin, or 0.5 nM Okadaic acid for 9 h. The cell lysates were harvested and subjected to Western blotting. **b** SH-SY5Y cells were treated with 50 μM 6-OHDA along with 0.5 nM Okadaic acid (Okad), 0.2 nM Cypermethrin (Cyper), or 10 μM Cyclosporin A (CsA) for 9 h and subjected to cell viability assay. Both Cypermethrin and Cyclosporin A treatment significantly blocked cell death, but Okadaic acid treatment did not block cell death (CTL: 100.0 ± 4.48 %, *n =* 6; 6-OHDA: 58.0 ± 0.74 %, *n =* 6; Okad: 37.6 ± 0.69 %, *n =* 6; Cyper: 86.6 ± 1.55 %, *n =* 6; CsA: 67.5 ± 0.90 %, *n =* 6, One-way ANOVA, *F*_4,29_ = 122.3, ****P < 0.0001, Newman-Keuls multiple comparison test: **P < 0.01, ***P < 0.001)

We tried to identify an upstream protein phosphatase (PP) which has an effect on PAK1 by oxidative stress. In order to address, the SH-SY5Y cells were treated with 6-OHDA together with Cantharidic acid for inhibition of PP2A and PP1, Cypermethrin for inhibition of PP2B, or Okadaic acid for inhibition of PP2A. The Cypermethrin treatment blocked the down-regulation of PAK1 activity, suggesting that PP2B might be an upstream phosphatase of PAK1 in the signaling induced by oxidative stress (Fig. 3a). Cyclosporin A, a widely used immunosuppressant, is also a potent inhibitor of PP2B, and it has been reported as a neuroprotective agent in experimental models of Parkinsonism [21]. Hence, in the subsequent experiment, we examined whether Cyclosporin A or Cypermethrin could rescue the viability of 6-OHDA treated cells. As expected, both Cyclosporin A and Cypermethrin could significantly rescue cell viability from oxidative stress (Fig. 3b).

### PAK1-DN expression decreases the level of Bcl-2 protein

**Fig. 4:**
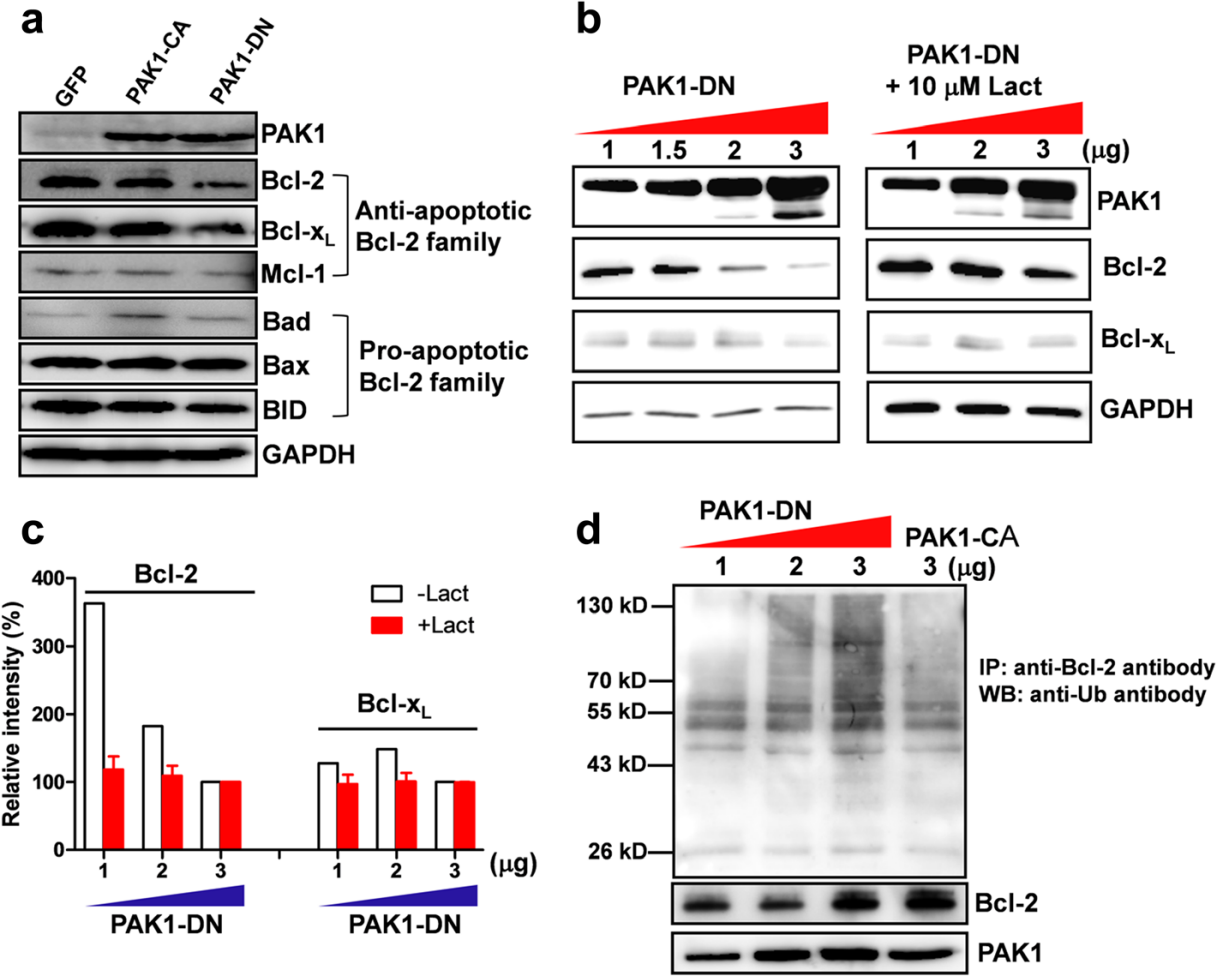
PAK1-DN expression reduces the Bcl-2 level. **a** PAK1-DN expression reduces level of anti-apoptotic Bcl-2 family proteins. Lysates of SH-SY5Y cells expressing GFP, PAK1-CA, or PAK1-DN were subjected to Western Blot analysis using antibodies against anti-apoptotic or pro-apoptotic Bcl-2 family proteins. **b** Reduction in Bcl-2 induced by PAK1-DN expression is mediated by ubiquitin-dependent protein degradation. HEK cells were transfected with 1, 1.5, 2, or 3 μg of PAK1-DN vector and incubated for 36 h and treated with 10 μM lactacystin or without for 12 h. The cell lysates were subjected to Western blot analyses using the indicated antibodies. **c** Quantification of Western blot analysis. To obtain relative values of Bcl-2 or Bcl-x_L_ level, band intensities from 1 and 2 μg expression of PAK1-DN was normalized by that from 3 μg expression of PAK1-DN in each blot. Reduction in Bcl-2 induced by dose-dependent expression of PAK1-DN reversed by lactacystin treatment [−Lact: 362.68 % (1 μg) → 182.6 % (2 μg) → 100.0 % (3 μg), +Lact: 118.5 ± 19.26 %, *n =* 3 (1 μg) → 109.05 ± 15.10 %, *n =* 3 (2 μg) → 100.0 %, *n =* 3 (3 μg)]. Bcl-x_L_ level was not altered [−Lact: 127.7 % (1 μg) → 148.4 % (2 μg:) → 100.0 % (3 μg), +Lact: 97.0 ± 13.89 %, *n =* 3 (1 μg) → 100.94 ± 12.59 %, *n =* 3 (2 μg) → 100.0 %, *n =* 3 (3 μg)]. **d** PAK1-DN expression increased the ubiquitination of Bcl-2 protein, but PAK1-CA expression did not increase the ubiquitination of Bcl-2 protein

Previous studies [10, 11] indicate that activation of PAK1 by IL3 directly or indirectly increase the phosphorylation level of Bad, a pro-apoptotic Bcl-2 family protein, consequently reducing the interaction between Bad and Bcl-2, and thereby increasing cell survival in FL5.12 lymphoid progenitor cells. In contrast, the autoinhibitory domain of PAK1 (amino acids 83–149) enhanced apoptotic cell death [11]. Additionally, the down-regulation of Bcl-2 level was correlated with the activity of PAK1 (Fig. 2). Thus, in order to identify the molecular mechanisms implicated in apoptotic cell death caused by down-regulation of PAK1 activity, we examined the level of the members of anti-apoptotic and pro-apoptotic Bcl-2 family proteins induced by PAK1-DN expression. As shown in Fig. 4a, Bcl-2 and Bcl-x_L_, members of anti-apoptotic Bcl-2 family proteins, were down-regulated by the PAK1-DN expression. Also, the down-regulation of proteins was recapitulated in the transient expression of PAK1-DN plasmid vector in human embryonic kidney (HEK) 293T cells (Additional file 4: Figure S4). Consequently, to identify the molecular mechanisms involved in the down-regulation, we examined the mRNA levels of Bcl-2 and Bcl-x_L_ by RT-PCR analysis. Interestingly, the decrease in the protein level by PAK1-DN expression was not correlated with the level of each mRNA, suggesting a regulatory mechanism in the post-translational stage (Additional file 5: Figure S5).

In order to further clarify whether the decrease of Bcl-2 or Bcl-x_L_ is a specific event regulated by PAK1-DN, we examined the dose-dependency of Bcl-2 decrease induced by PAK1-DN. The HEK 293T cells were transfected with 1, 1.5, 2, or 3 μg of PAK1-DN plasmids, and subsequently the protein levels of Bcl-2 or Bcl-x_L_ were examined. As shown in Fig. 4b, c, the level of Bcl-2 was inversely correlated with the level of PAK1-DN. The decrease in Bcl-x_L_ was only evident in cells transfected with 3 μg of PAK1-DN plasmids, but it did not show any dose-dependency. In the subsequent experiments, we examined the levels of Bcl-2 and Bcl-x_L_ induced by the expression of PAK1-WT, PAK1-CA, or other inactive-form mutants (PAK1^H83,86L^, PAK1^K299R^, PAK1 67–150 amino acids). No alteration in the Bcl-2 level was detected, except PAK1^K299R^ or PAK1 67–150 aa expression. The levels of Bcl-x_L_ induced by transient expression of PAK1 mutants were not altered in all results, being different from those induced by stable expression of PAK1-DN (Additional file 6: Figure S6).

Previous studies have reported that the cellular level of Bcl-2 is modulated by the ubiquitin/proteasome-dependent pathway and this degradation is dependent on the phosphorylation status of Bcl-2, which is modulated by mitogen-activated protein (MAP) kinase [22, 23]. Hence, we examined whether the down-regulation of Bcl-2 by PAK1-DN is dependent on the ubiquitin/proteasome-dependent pathway. As shown in Fig. 4b, c, the down-regulation of Bcl-2 level by PAK1-DN was completely blocked by 10 μM lactacystin, an inhibitor of the ubiquitin/proteasome-dependent pathway. In addition, treatment with MG-132, another specific inhibitor to the ubiquitin/proteasome-dependent pathway, showed the same results (data not shown). Consequently, in the subsequent experiment, we examined the ubiquitination of Bcl-2 induced by PAK1-DN expression. Indeed, PAK1-DN expression predominantly increased the ubiquitination level of Bcl-2 protein (Fig. 4d). These results suggest that PAK1 activity could modulate the Bcl-2 level via the ubiquitin/proteasome-dependent mechanism.

### Bcl-2 level could be modulated by both PAK1-MAPK signaling and PAK1 directly

**Fig. 5:**
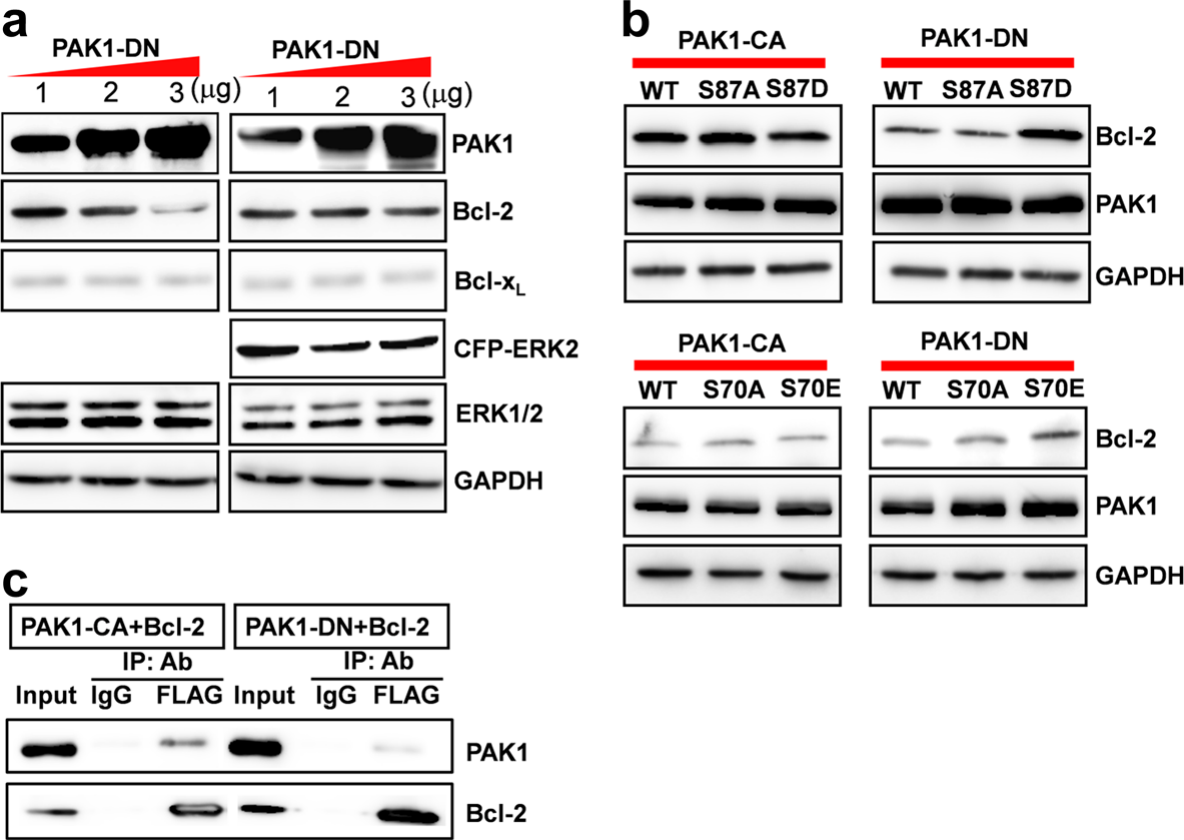
Reduction in the Bcl-2 level induced by PAK1-DN is mediated by PAK1-ERK or PAK1 directly. **a** HEK 293T cells were transfected with 1, 2, or 3 μg of PAK1-DN vectors and with 1 μg of CFP-ERK2 or without CFP-ERK2. Dose-dependent reduction in the Bcl-2 level induced by PAK1-DN was blocked by ERK2 expression. **b** Bcl-2^S87D^ (serine 87 → aspartic acid), a phosphomimetic form by ERK was resistant to PAK1-DN expression. Bcl-2^S87^ (serine 87 → alanine) did not show resistance to PAK1-DN expression. Bcl-2^S70E^ (serine 70 → glutamic acid), a putative phosphomimetic form by PAK1 was also resistant to PAK1-DN expression. All Bcl-2 mutants were not altered by PAK1-CA expression. **c** HEK 293T cells were transfected with FLAG-tagged Bcl-2 and Myc-tagged PAK1-CA or PAK1-DN vectors. The cell lysates were immunoprecipitated with anti-FLAG antibody and the resulting immunoprecipitates were subjected to Western blot analysis using anti-Myc antibody

As previously described, Bcl-2 protein is down-regulated by the ubiquitin/proteasome-dependent mechanism and this degradation is dependent on the phosphoryation status of Bcl-2, which is modulated by the mitogen-activated protein kinase (MAPK) pathway [22, 23]. Hence, we examined whether the down-regulation of Bcl-2 by PAK1-DN expression could be rescued by extracellular signal-regulated kinase (ERK) expression. As a result, the down-regulation of Bcl-2 by PAK1-DN expression was recovered by ERK2 expression (Fig. 5a). In order to further clarify this result, serine 87 on Bcl-2, which is reported to be a major site for phosphorylation by MAP kinase, was changed to aspartic acid, mimicking phosphorylation (Bcl-2^S87D^, S87D) or an alanine residue (Bcl-2^S87A^, S87A). As expected, the level of S87D was not altered by PAK-DN expression. These results suggest that the Bcl-2 level could be modulated by the PAK1-MAPK pathway.

We questioned whether the level of Bcl-2 might be directly regulated by PAK1. A putative site for phosphorylation by PAK1 on the amino acid sequences of human Bcl-2 was examined using GPS 2.1.2 (Group-based Prediction system), a kinase-specific prediction program [24]. Serine 70 was isolated as a putative site for phosphorylation by STE20/PAK1. We constructed mutants in which serine 70 was changed to glutamic acid (Bcl-2^S70E^, S70E) or alanine (Bcl-2^S70A^, S70A), and we examined the level of each Bcl-2 mutant induced by PAK1-DN expression. Interestingly, S70E was resistant to the degradation mediated by PAK1-DN expression (Fig. 5b). In order to examine the association of PAK1 with Bcl-2, we performed coimmunoprecipiation (co-IP) assay. PAK1-CA was associated with Bcl-2 immunecomplexes and this association was much stronger than that of PAK1-DN with Bcl-2 immunecomplexes (Fig. 5c). Taken together, these data suggest that Bcl-2 level could be modulated by PAK1-MAPK signaling and/or PAK1 directly.

### PAK1-CA recovers behavioral and cellular defects in hemiparkinsonian rat model

**Fig. 6:**
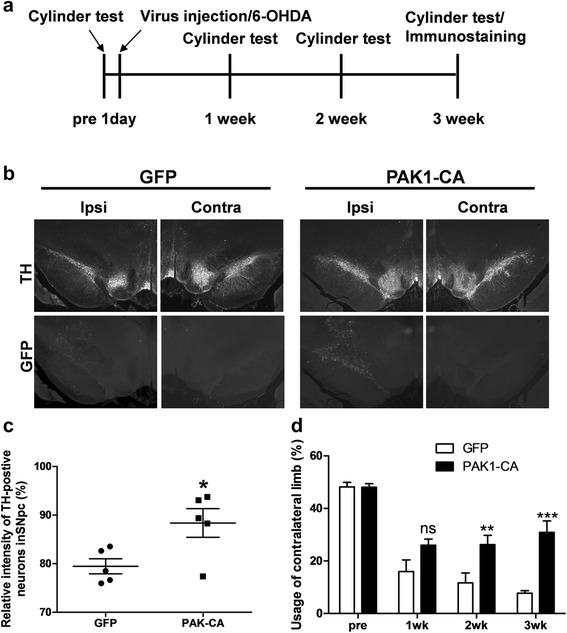
PAK1-CA expression recovers behavioral defect and loss of mesencephalic dopamine neurons. **a** Experimental scheme and procedure. **b** Lentiviruses encoding GFP or PAK1-CA were infused into the right SNpc at rate of 0.5 μl/min at the following coordinates: anteroposterior, −5.3 mm from the bregma; mediolateral, 2.3 mm; dorsoventral, −7.3 mm below surface of the dura. After 3 weeks, the midbrain tissue sections were immunostained with anti-Tyrosine Hyroxylase antibody and visualized by immunostaining using Cy3-conjugated goat anti-rabbit IgG antibody. **c** The loss of DA neurons in the SNpc induced by 6-OHDA treatment was blocked by PAK1-CA expression, but not by GFP expression (GFP: 79.5 ± 1.55 %, *n =* 5, compared to the ipsilateral side; PAK1-CA: 88.4 ± 2.94 %, *n =* 5, compare to ipsilateral side, Student’s *t*-tests, *P < 0.05). **d** PAK1-CA expression significantly improved usage of the contralateral limb in a hemiparkinsonian animal model (GFP: PAK1-CA, pretest, 48.2 ± 1.69, *n =* 5: 48.08 ± 1.37, *n =* 8; 1 week, 16.0 ± 4.46, *n =* 5: 26.00 ± 2.30, *n =* 8; 2 weeks, 11.7 ± 3.73, *n =* 5: 26.21 ± 3.58, *n =* 8; 3 weeks, 7.8 ± 0.93, *n =* 4: 30.88 ± 4.32, *n =* 6; Two-way ANOVA, *F*_3,41_ = 39.40, ***P < 0.0001, Bonferroni multiple comparison tests, ns: not significant, **P < 0.01, ***P < 0.001)

The catecholamine neurotoxin, 6-hydroxydopamine (6-OHDA) has been widely used to PD model [25, 26]. We examined the effect of PAK1-CA expression on the DA neuron survival and animal behavior in a 6-OHDA-induced hemiparkinsonian rat model. In order to induce progressive death of nigral neurons, 6-OHDA was injected into the striatum, and subsequently, lenti-PAK1-CA or lenti-GFP virus was injected into the ipsilateral substantia nigra pars compacta (SNpc) [27, 28]. During 3 weeks, behavioral assay was performed to examine forelimb-use asymmetry at every week (Fig. 6a) [25, 29, 30]. Finally, tyrosine hydroxylase (TH)-positive neurons in the ipsilateral SNpc were measured by comparing with TH-positive neurons in the contralateral SNpc. As shown in Fig. 6b, c, PAK-CA expression in the substantia nigra significantly blocked the loss of TH-positive neurons induced by 6-OHDA treatment. Consistent with cellular protection, the PAK1-CA expression significantly recovered behavioral defects from 2 weeks after the infusion and infection, compared to those in the GFP-expressing control group. Additionally, the recovery from loss of TH-positive neurons and behavioral defects by the PAK1-CA expression was recapitulated in 1-Methyl-4-phenyl-1,2,3,6-tetrahydropyridine hydrochloride (MPTP)-induced hemiparkinsonian rat model (Additional file 7: Figure S7). These data strongly suggest that the activity of PAK1 could be related with the survival of TH-positive neurons and behaviors.

## Discussion

**Fig. 7:**
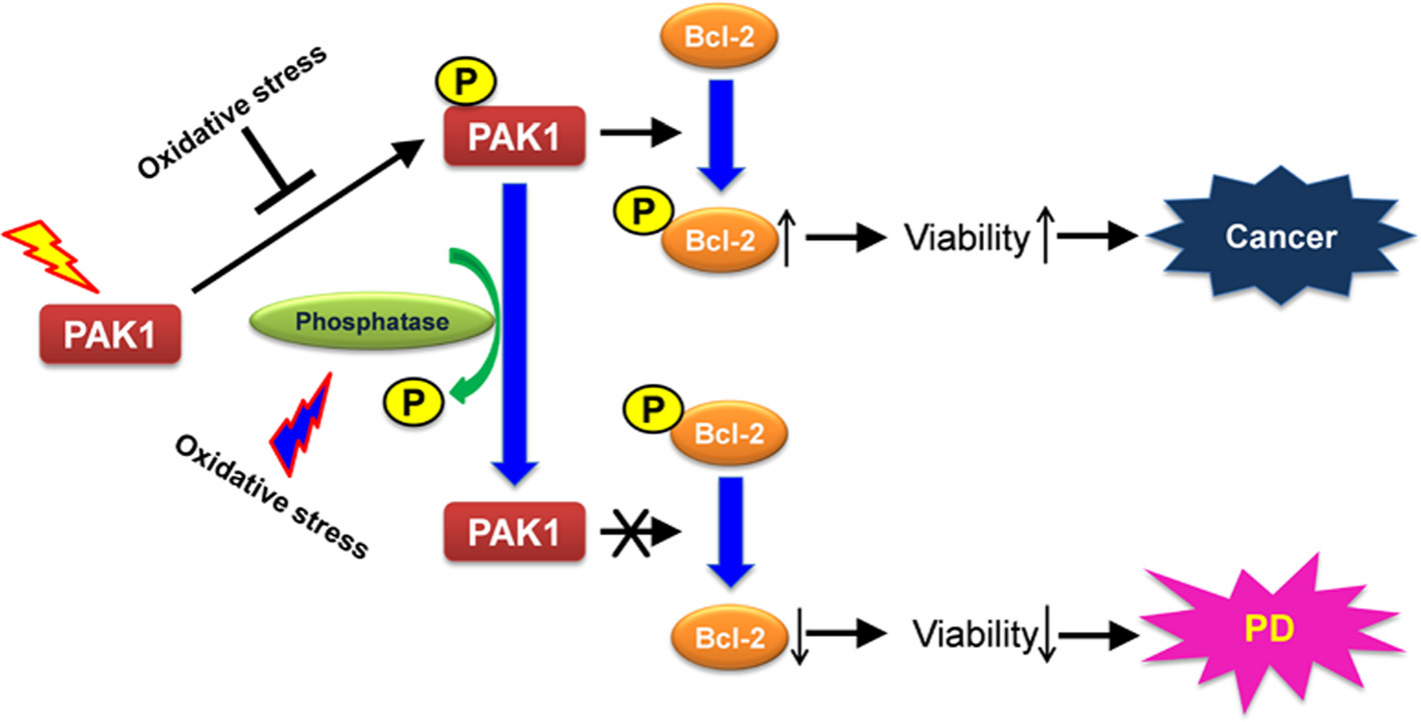
Model of PAK1 roles in the pathogenesis of diseases. An oxidative stress might induce activation of a phosphatase for PAK1 or inhibit phosphorylation of PAK1 via other mechanisms. Consequently, the down-regulation of PAK1 activity decreases the phosphorylation of Bcl-2 and the dephosphorylated Bcl-2 proteins are to be more susceptible to an ubiquitin/proteasome-dependent degradation. Reduction of Bcl-2 level induces loss of cells, such as mesencephalic dopamine neurons, thereby inducing Parkinson’s disease (PD)

Since a very long time, there have been many discussions on the inverse comorbidity between some cancers and neurodegenerative diseases [17]. A genetic determinant can decide the fate of cells, depending on the situations or the characteristic of cells [18]. Previous studies have described that overactivation or overexpression of PAK1 is strongly correlated with cancers [4, 5]. In our study, down-regulation of PAK1 activity, and not amount, was correlated with cell death and that occurs at the initial stage of cell death induced by oxidative stress, such as 6-OHDA treatment (Fig. 1, 2). These results suggest that PAK1 might function as a molecular determinant that may decide the direction for development of neurodegenerative diseases, such as Parkinson’s disease (Fig. 7).

It has been widely accepted that apoptosis (type I cell death) is the major cause of neuronal loss in PD [31]. Some studies, using postmortem brain sections of PD patients, have reported that loss of the nigral neurons is due to apoptosis induced by an increase in Bax (a proapoptotic member of the Bcl-2 family)-dependent mitochondrial membrane permeability [31–33]. If cell fate is determined by the ratio of pro-apoptotic to anti-apoptotic proteins, a reduction in Bcl-2 could increase Bax-triggered apoptosis. Of course, some part of this apoptotic cell death might originate from dephosphorylation of Bad induced by inactivation of PAK signaling [10, 11]. However, if we consider that the expression of Bax in the melanized nigral neurons is more related with PD brains [32, 33], Bax-triggered apoptosis induced by the reduction in Bcl-2 could be a major pathway. Even in our conclusion, the possibility that two pathways act synergistically or simultaneously, cannot be excluded. Thus, in order to clarify this, more studies are needed.

Unwanted proteins should be cleared by cellular clearance systems. The failure of clearance system could increase the number of misfolded and aggregated proteins. Thus, the ubiquitin-proteasome system is closely linked with the development of PD. Indeed, mutations of genes involved in the ubiquitin-proteasome system are found in several types of familial PD and proteasomal enzymatic activity is reduced in sporadic PD [34]. In our study (Fig. 2, 3) and previous reports [22, 23], oxidative stress regulated PAK1 activity. Subsequently, PAK1 could regulate the level of Bcl-2 protein through the ubiquitin-proteasome system by modulating its phosphorylation status through the extracellular signal-regulated kinase (ERK) pathway [22, 23]. However, these mechanisms only work when ubiquitin-proteasome system is intact or partially working. Protein aggregates, known as Lewy bodies, in nigral neurons have toxic effects. Production of protein aggregates might be due to an imbalance between the amount of unwanted proteins and the capacity to remove them. Indeed, the reduction of removal capacity is correlated with aging [35, 36].

Extracellular signals that lead to the activation or inactivation of PAK1 have not been clearly elucidated. In particular, inactivation mechanisms including dephosphorylation mechanisms still remain to be elucidated. Although PP2A [37, 38], POPX1/2 [39] and PP2Cα [40] have been reported as PAK1 regulators in different cellular contexts, there has been no report on the phosphatase for PAK1 in response to oxidative stress. Our data suggest calcineurin, PP2B, as a phosphatase for PAK1 in the signaling involved in oxidative stress (Fig. 3a). Indeed, the inhibitors of PP2B decreased the death rate of neuroblastoma cells induced by 6-OHDA treatment (Fig. 3b), suggesting a new target for PD therapy. For treatment of PD, levodopa (L-dopa), a dopamine precursor has been widely used. However, long-term treatment causes some side effects such as psychiatric symptoms, wearing-off phenomenon, or on/off phenomenon. The focus of new therapy has been on the development of agents that suppress or delay neuronal death [21]. Cyclosporin A, an immunosuppressant, has been used to suppress the immune response after transplantation. Interestingly, treatment of Cyclosporin A protects cortical neurons from traumatic brain injury [41, 42] and attenuates the decrease of tyrosine hydroxylase in the nigrostriatal DA neurons and DA neurons itself in a 6-OHDA animal model of PD [21]. Our data also showed that treatment with Cyclosporin A increased the viability of neuroblastoma cells treated with the neurotoxin, 6-OHDA (Fig. 3). Thus we suggest a novel molecular mechanism for neuroprotective effects of Cyclosporin A. Inactivation of PAK1 induced by oxidative stress might be blocked by the inhibition of PP2B by Cyclosporin A, thereby increasing cell viability (Fig. 7). However, more studies are required for the clinical application of Cyclosporin A in PD patients.

## Methods

### Experimental animal

Adult female (~230 g) and postnatal 1-day-Sprague–Dawley rats were purchased from SAMTAKO (Osan, Korea) for the animal model of Parkinson’s disease and the culture of dopamine neurons, respectively. All experiments were performed in accordance with the approved animal protocols and the guidelines of the Institutional Animal Care and Use Committee of Chungbuk National University (CBNUA-432-12-02).

### Cell culture

Human embryonic kidney (HEK) 293T cells were cultured in Dulbecco’s modified Eagle’s medium (DMEM, GIBCO^®^, Grand Island, NY, USA) supplemented with 7–10 % fetal bovine serum (GIBCO^®^) and supplied with 5 % CO_2_, 37 °C. Human neuroblastoma cells (SH-SY5Y) were grown in Eagle’s Minimum Essential Medium and F12 Medium supplemented with 10 % fetal bovine serum (GIBCO^®^). To obtain culture of mesencephalic dopamine neurons, astrocytes were placed on collagen/poly-L-lysine coated coverglass and incubated for 7 days. Dopamine neurons isolated from the substantia nigra (SN) and ventral tegmental area (VTA) of post-natal 1-day-old rats were placed on the astrocyte monolayer and incubated for 16 days before use [43].

### DNA construction and lentivirus

For expression of PAK1 and its mutants, PAK1 wild type (WT), kinase-active PAK1^T423E^ (PAK1-CA) and dominant-negative PAK1^H83,86L, K299R^ (PAK1-DN) [44] were inserted into the pCMV-myc vector (Clontech, Palo Alto, CA, USA) for cell line expressions, and into the pHJEF1a(sin) vector for lentiviral expression. For expression of ERK2, ERK2 was inserted into the pCFP-C1 vector (Clontech), resulting in pCFP-ERK2. The preparations of lentivirus and Sindbis virus were performed according to the previously described methods [45]. Human Bcl-2 mutants (S70E, S70A, S87D, and S87A) were made by recombinant PCR methods using the following primer sets: Bcl-2-S70E-S: 5′-aggaccgagccgctgcagaccccggct-3′, Bcl-2-S70E-A: 5′-tgcagcggctcggtcctggcgaccggg-3′, Bcl-2-S70A-S: 5′-aggaccgcgccgctgcagaccccggct-3;, Bcl-2-S70A-A: 5′-tgcagcggcgcggtcctggcgaccggg-3′, Bcl-2-S87D-S: 5′-gcgctcgacccggtgccacctgtggtcca-3, Bcl-2-S87D-A, 5′-ggcaccgggtcgagcgcaggccccgcggc-3′, Bcl-2-S87A-S: 5′-gcgctcgccccggtgccacctgtggtcca-3′, Bcl-2-S87A-A: ggcaccggggcgagcgcaggccccgcggc-3′.

### Stable cell lines

To obtain pure cell cultures expressing GFP, PAK1 wild type (WT), constitutively active PAK1^T423E^ (PAK1-CA) mutant, or dominant-negative PAK1^H83,86L, K299R^ (PAK1-DN) mutant, after lentiviral infection of encoding GFP only or each PAK1 mutant and bicistronically linked GFP, SH-SY5Y cells were sorted by a fluorescence-activated cell sorter (FACSCalibur, BD Biosciences, Franklin Lakes, NJ, USA) using fluorescence of GFP, and stored.

### Cell viability assay and TUNEL staining

For the viability assay, SH-SY5Y cells expressing GFP, PAK1-WT, PAK1-CA or PAK1-DN, were plated at 1X10^5^ cells per well and incubated for 72 h. For assessing viability following oxidative stress, cells were plated and incubated for 24 h and additionally incubated with 700 μM hydrogen peroxide (Sigma-Aldrich, St Louis, MO, USA), 100 μM 6-hydroxydopamine (Sigma-Aldrich), or without both for 12 h. For the viability assay after phosphatase inhibitor treatment, SH-SY5Y cells (1X10^5^ cells) were pretreated with 0.2 nM Cypermethrin (Santa Cruz, Santa Cruz, CA, USA), 0.5 nM Okadaic acid (Sigma-Aldrich), or 10 μM Cyclosporin A (Tocris, Bristol Avonmouth, UK) for 30 min and subsequently treated along with 50 μM 6-OHDA for 8 h 30 min. The number of viable cells was determined by colorimetric assay using WST-8 (Dojindo Laboratories, Kumamoto, Japan). To measure apoptotic cell death, at 72 h after cell plating, cultures were subjected to TUNEL staining using *In Situ* Cell Death Detection kit (Roche, Basel, Switzerland). The apoptosis rates were determined by the number of TUNEL-positive cells in that of GFP-positive cells on three culture dishes for each condition.

### Western blotting

SH-SY5Y cells expressing GFP, PAK1-CA, or PAK1-DN were plated and incubated for 72 h. The cultured HEK 293T cells were transfected with the indicated plasmids and GFP vector, thus making up the total amount of plasmids used in transfection to 3 μg, and were incubated 48 h to induce expression. For lactacystin (Tocris) treatment, after 36 h of incubation, transfected cells were treated with 10 μM lactacystin for 12 h. For Western blot analysis, the lysates of cells were prepared by adding the lysis buffer (150 mM NaCl, 1 % IGEPAL^®^ CA-630, 50 mM Tris⋅Cl [pH 8.0]), separated on the SDS-PAGE and transferred to PVDF membrane (Millipore, Billerica, MA, USA). The blots were incubated with the indciated antibody. For control, the blot was washed with stripping buffer (Thermo Scientific, Rockford, IL, USA) and re-probed. Antibodies used in Western blot analyses were as follows: anti-FLAG antibody (M2, F1804, Sigma-Aldrich), anti-c-Myc-antibody (9E10, M4493, Sigma-Aldrich), anti-Bcl-2 antibody (50E3, #2870, Cell Signaling, Danvers, MA, USA), anti-Bcl-x_L_ antibody (54H6, #2764, Cell Signaling), anti-Mcl-1 antibody (#4572, Cell Signaling), anti-Bad antibody (#9292, Cell Signaling), anti-Bax antibody (#2772, Cell Signaling), anti-BID antibody (#2002, Cell Signaling), anti-PAK1 polyclonal antibody (N-20, sc-882, SANTA CRUZ Biotechnology), anti-phosphorylated PAK1 antibody (Thr 423, sc-21903-R, SANTA CRUZ Biotechnology), anti-ERK1/2 antibody (#9102, Cell Signaling), phosphorylated ERK2 (Thr202/Tyr204, # 9101, Cell Signaling), anti-tyrosine hydroxylase antibody (AB152, Millipore, Billerica, MA, USA).

### Ubiqutination assay

HEK cells were transfected with the indicated amount of PAK1-DN or PAK1-CA plasmids, incubated for 36 h, treated with 10 μM lactacystin (Tocris) for 12 h and subjected to immunoprecipitation with anti-Bcl-2 antibody (Cell Signaling). Immunoprecipitates were subjected to Western blot analysis using anti-monoclonal mono- and polyubiquitinylated conjugates antibody (FK2, BML-PW8810, Enzo Life Sciences, Farmingdale, NY, USA).

### Immunostaining

Cultured neurons were treated with 100 μM of 6-OHDA for 9 h, fixed with 4 % paraformaldehyde solution. Immunocytochemistry was conducted as described in a previous study [46]. For immunohistochemistry, anesthetized animals were dissected and fixed by vascular perfusion with 4 % paraformaldehyde solution through the heart. Total brain was isolated and additionally incubated with 4 % paraformaldehyde solution for 24 h. Then brain tissues were incubated with 20 % sucrose solution for 36 – 48 h, mounted in OCT embedding compound, frozen at −80 °C, and coronally cut into 30 μm thick tissue sections by a cryostat (Leica 1650, Leica, Wetzlar, Germany). Neuronal cultures and tissue sections were permeabilized with PBST (0.1 % Triton X-100, 0.2 % BSA, 1X PBS, pH7.4), and blocked with a preblocking agent (2 % BSA, 0.08 % Triton X-100, 1X PBS, pH7.4). The cultures were treated with PAK1 or p-PAK1 antibody and visualized by secondary staining with Cy3-conjugated goat anti-rabbit IgG antibody (111–165–144, Jackson ImmunoResearch Laboratories, West Grove, PA). Tissue sections were treated with anti-Tyrosine Hydroxylase antibody (Millipore) and subsequently immunostained with Cy3-conjugated goat anti-rabbit IgG antibody. Immunostained cells and tissue sections were acquired by an up-right fluorescence microscope (BX-51, Olympus, Tokyo, Japan) equipped with a CCD camera (DP30BW, Olympus) and the images were analyzed by NIH image analysis program (ImageJ ver 1.47v, Bethesda, MA, USA).

### Phosphatase inhibitor assay

SH-SY5Y cells were pretreated with 200 nM Cantharidic acid (Santa Cruz Biotechnology), 0.2 nM Cypermethrin (Santa Cruz Biotechnology), or 0.5 nM Okadaic acid (Sigma-Aldrich) for 30 min and treated along with 50 μM 6-OHDA for 8 h 30 min. The cell lysates were subjected to Western blot analysis.

### Stereotaxic surgical procedure

Adult female Sprague–Dawley rats (~230 g) were anesthetized by a subcutaneous injection of a Zoletil/Rompun cocktail solution (5 mg/kg Zoletil 50, Virbac Laboratories, Carros, France; 0.5 ml/kg Rompun, Bayer, Berlin, Germany). Animals were placed into a stereotaxic frame (ASI^®^ Instruments, Warren, MI, USA). Burr holes were drilled to allow a single unilateral injection of 6-OHDA or a double injection of 6-OHDA or saline and lentivirus. A 4 μl dose of 6-OHDA (5 μg/μl) was infused into the right striatum at a rate of 0.5 μl/min at the following coordinates: anteroposterior, 1.0 mm from the bregma; mediolateral, −3.0 mm; dorsoventral, −5.0 mm below surface of the dura [27]. A 2 μl dose of lentivirus encoding GFP or PAK1-CA (1–2 X10^7^ TU/ml) was infused into the right SNpc at a rate of 0.5 μl/min at the following coordinates: anteroposterior, −5.3 mm from the bregma; mediolateral, −2.3 mm; dorsoventral, −7.3 mm below surface of the dura [27].

### Behavioral test (cylinder test)

A unilateral lesion of the nigrostriatal pathway was measured by examining the asymmetry in forelimb usage [29, 30]. Experimental animals were placed in a transparent cylinder, 30 cm high and 20 cm in diameter, for 5 min. The contact with the wall by the impaired, unimpaired or both forelimbs was scored and presented as percentage. Animals which showed usage of the left or right forelimb in a pretest up to more than 70 % were excluded in subsequent experiments.

### Statistical analysis

All data are presented as mean ± SEM. Statistical significance between the two groups was measured by Student’s *t*-tests and that among more than three groups by ANOVA analysis with an appropriate post-hoc test. Statistical difference in cell viability induced by PAK1 and its mutants was analyzed by one-way ANOVA and Newman-Keuls multiple comparison test. Animal behavior was analyzed by two-way ANOVA and Bonferroni multiple comparison test.

### Ethics approval and consent to participate

All animal experiments were performed in accordance with the approved animal protocols and the guidelines of the Institutional Animal Care and Use Committee of Chungbuk National University (CBNUA-432–12–02).

### Consent for publication

Not applicable.

## Conclusions

These results indicate the roles of PAK1 in neuronal death and neurodegeneration induced by oxidative stress. Down-regulation of PAK1 activity was strongly correlated with cell death and loss of mesencephalic DA neurons. Conversely, up-regulation of PAK1 activity recovered loss of mesencephalic DA neurons and behavioral defects in a hemiparkinsonian animal model. Taken together, our data provided novel mechanisms in the pathogensis of PD and a useful therapeutic target.

### Availability of data and materials

The datasets supporting the conclusions of this article are included within the article.

## Additional files

Additional file 1: Figure S1. SH-SY5Y cells and HEK 293T cells expressed endogenous PAK1. The 10 or 20 μg of lyastes from each cell line was separated on SDS-PAGE and subjected to Western blot analysis using anti-PAK1 antibody. The membrane was deprobed and reprobed by anti-GAPDH antibody. (TIF 2426 kb) Additional file 2: Figure S2. Reduction of Bcl-2 level by 6-OHDA treatment is independent of transcription. a. SH-SY5Y cells were treated 50 μM 6-OHDA for 9 h and total RNA was isolated. Purified 3 μg of total RNA was used in RT-PCR analysis for Bcl-2, Bcl-_XL_, and GAPDH mRNA (w/o: negative control without RT reaction). b. Bar graphic representation of RT-PCR analysis. The intensity of Bcl-2 or Bcl-_XL_ band at each time point was normalized by the intensity of GAPDH band, respectively. The data was presented a relative value to value of 0 h. (TIF 163 kb) Additional file 3: Figure S3. 6-OHDA treatment reduces phosphorylated PAK1 level in cultured DA neurons. a. Mesencephalic DA neurons were isolated from the ventral tegumental area (VTA) and cultured for 16 day before use. Cultured DA neurons were treated with 100 μM 6-OHDA for 9 h and fixed. The cultures were immunostained with anti-monoclonal tyrosine hydroxylase antibody (TH-16, T2928, Sigma-Aldrich), anti-polyclonal PAK1 antibody (N-20, sc-882, SANTA CRUZ Biotechnology), or anti-phospho-PAK1 antibody (sc-21903-R, SANTA CRUZ Biotechnology). The cultures were subsequently immunostained with Alex Fluor® 488 anti-mouse IgG antibody (A11029, Molecular Probes) for visualization of TH staining and Cy3-conjugated anti-rabbit IgG antibody (111–165–144, Jackson ImmunoResearch Lab) for PAK1 and p-PAK1 staining. Images were acquired by fluorescent microscopy (BX-51, Olympus) and analyzed by NIH image analysis program (ImageJ ver 1.47v). Scale bar: 20 μm. b. Bar graphic representation of image analysis. 6-OHDA treatment could not change PAK1 level (CTL: 100.0 ± 7.40 %, *n =* 55 neurons from 6 plates; 6-OHDA: 109.2 ± 3.79 %, *n =* 56 neurons from 6 plates, ns: not significant) but decreased p-PAK1 level (CTL: 100.0 ± 5.56 %, *n =* 20 neurons from 3 plates; 6-OHDA: 72.71 ± 8.81 %, *n =* 27 neurons from 3 plates, Student’s *t*-tests: * *P* < 0.05) (TIF 10356 kb) Additional file 4: Figure S4. PAK1-DN expression reduces the Bcl-2 level in HEK 293T cells. a. Lysates of HEK 293T cells transfected with GFP, PAK1-WT, PAK1-CA, or PAK1-DN vectors were subjected to Western blot analysis using Bcl-2 antibody. b. Quantification of Western blot analysis. Each band was normalized with the band intensity of WT (GFP: 101.0 ± 5.19 %, *n =* 5; WT: 100.0 %, *n =* 5; CA: 95.9 ± 6.46 %, *n =* 5; DN: 73.16 ± 6.87 %, *n =* 5; One-way ANOVA, *F*_3,19_ = 5.909, ^**^ *P* = 0.0065, Newman-Keuls multiple comparison test: ^**^ *P* < 0.01, ^*^ *P* <0.5). (TIF 2613 kb) Additional file 5: Figure S5. Reduction of Bcl-1 by PAK-DN is independent of transcription. a. Total RNA was purified from SH-SY5Y cells expressing GFP, PAK1-CA, or PAK1-DN. And 3 μg of total RNA from each was used for RT-PCR analysis using SuperScript III (Invitrogen). The PCR primers used in RT-PCR are follows; Bcl-2 forward: 5′-agatgggaacactggtggag-3′, Bcl-2 reverse: 5′-cttccccaaaagaaatgcaa-3′, Bad forward: 5′-cctcaggcctatgcaaaaag-3′, Bad reverse: 5′-taaacctggctcgcgactta-3′, Bcl-x_L_ forward: 5′-ggctgggatacttttgtgga-3′, Bcl-x_L_ reverse: 5′-gggagggtagagtggatggt-3′, GAPDH forward: 5′-gagtcaacggtttggtcgt-3′, GAPDH reverse: 5′-ttgattttggagggatctcg-3′. b. Bar graphic representation of RT-PCR analysis. Each band was normalized with intensity of GAPDH. (TIF 208 kb) Additional file 6: Figure S6. Other mutants of PAK1- inactive form reduce Bcl-2 level. a. HEK cells were transfected with 1, 2, or 3 μg of GFP, PAK1-WT, PAK1-H83,86L, or PAK1-K299R vectors and incubated for 48 h. The cell lysates were subjected to Western blotting analysis using anti-Myc, Bcl-2, Bcl-x_L_, or GAPDH antibody. b. The autoinhibitory domain (AID) of hPAK1 (67–150 amino acids) were amplified by PCR methods (hPAk1-67-R1-S: 5′-ggaattcggaagaaagagaaagagcggc-3′, hPAK1-150-Xho-A: 5′-ccgctcgagttaagctgacttatctgtaaagc-3′) and inserted to EcoRI/XhoI site of pCMV-Myc vector (Clontech) in-frame with Myc-tag, producing PAK1-DN (67–150) [47]. HEK cells were transfected with 1, 2, or 3 μg of PAK1-DN (67–150) vector and cell lysates were analyzed by Western blotting assay. (TIF 278 kb) Additional file 7: Figure S7. PAK1-CA expression rescues behavioral defect and loss of mesencephalic dopamine neurons induced by MPTP injection. a. Experimental scheme and procedure. b. Lentiviruses encoding GFP or PAK1-CA were infused into the right SNpc at rate of 0.5 μl/min at the following coordinates: anteroposterior, −5.3 mm from the bregma; mediolateral, −2.3 mm; dorsoventral, −7.3 mm below surface of the dura. A 2 μl dose of MPTP-HCl (1 μM, 1-Methyl-4-phenyl-1,2,3,6-tetrahydropyridine hydrochloride, Sigma-Aldrich) was infused into the right striatum at a rate of 0.5 μl/min at the following coordinates: anteroposterior, 1.0 mm from the bregma; mediolateral, −3.0 mm; dorsoventral, −5.0 mm below surface of the dura [48]. After 3 weeks, the midbrain tissue sections were immunostained with anti-Tyrosine Hyroxylase antibody and visualized by immunostaining using Cy3-conjugated goat anti-rabbit IgG antibody. c. The loss of DA neurons in the SNpc induced by MPTP injection was blocked by PAK1-CA expression, but not by GFP expression (GFP: 75.7 ± 0.41 %, *n =* 3, compared to the ipsilateral side; PAK1-CA: 101.4 ± 2.71 %, *n =* 4, compare to ipsilateral side, Student’s *t*-tests, ***P* < 0.01). d. PAK1-CA expression significantly improved usage of the contralateral limb in a hemiparkinsonian animal model (GFP: PAK1-CA, pretest, 48.8 ± 1.59, *n =* 3: 52.8 ± 1.92, *n =* 4; 1 week, 29.6 ± 2.50, *n =* 3: 44.03 ± 5.18, *n =* 4; 2 weeks, 34.2 ± 3.30, *n =* 3: 48.30 ± 2.58, *n =* 4; 3 weeks, 28.97 ± 1.09, *n =* 3: 45.75 ± 2.15, *n =* 4; Two-way ANOVA, *F*_3, 20_ = 1.746, *P* = 0.19, 1, Bonferroni multiple comparison tests, **P* < 0.05, ***P* < 0.01). (TIF 2786 kb)

## Abbreviations

PAK1: p21-activated serine/threonine kinase 1
DN: dominant-negative form
CA: constitutively active form
Bcl-2: B-cell lymphoma 2
ERK: extracellular signal-regulated kinase
DA: dopamine
SN: substantia nigra
Bcl-xL: B-cell lymphoma-extra large
Mcl-1: myeloid cell leukemia 1
Bad: Bcl2-associated agonist of cell death
Bax: Bcl2-associated X protein
Bak: Bcl-2 homologous antagonist/killer
Bok: Bcl-2 related ovarian killer
Bid: Bcl-2 interacting domain
IL3: interleukin 3
AD: Alzheimer’s disease
HD: huntington’s disease
Rac: Ras-related C3 botulinum toxin substrate
PD: parkinson’s disease
RT-PCR: reverse transcription-polymerase chain reaction, MAPK, mitogen-activated protein kinase
co-IP: co-immunoprecipitation
6-OHDA: 6-hydroxydopamine
PP: phosphatase
TH: tyrosine hydroxylase
SNpc: substantia nigra pars compacta
POPX1: partner of pix 1
HEK: human embryonic kidney
VTA: ventral tegmental area
CFP: cyan fluorescent protein
TUNEL: terminal deoxynucleotidyl transferase dUTP nick end labeling
OCT: optimum cutting temperature
Cy3: cyanine 3
BSA: bovine serum albumin
CCD: charge-coupled device
TMR: tetramethylrhodamine
SDS-PAGE: sodium dodecyl sulfate polyacrylamide gel electrophoresis
PVDF: polyvinylidene fluoride
SEM: standard error of the mean
ANOVA: analysis of variance
MPTP: 1-Methyl-4-phenyl-1,2,3,6-tetrahydropyridine
