# Supplementary figures and images for: Down-regulation of p21-activated serine/threonine kinase 1 is involved in loss of mesencephalic dopamine neurons
Source: Mol Brain. 2016 Apr 27;9:45. doi: 10.1186/s13041-016-0230-6 (PMC4848805; doi:10.1186/s13041-016-0230-6)

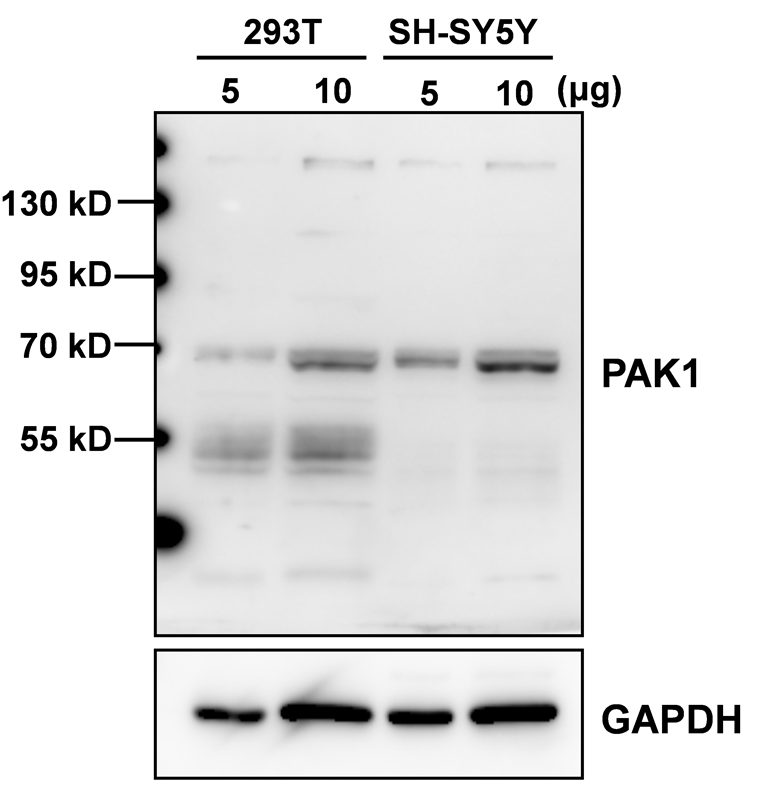

Supplement: Additional file 1: Figure S1. — SH-SY5Y cells and HEK 293T cells expressed endogenous PAK1. The 10 or 20 μg of lyastes from each cell line was separated on SDS-PAGE and subjected to Western blot analysis using anti-PAK1 antibody. The membrane was deprobed and reprobed by anti-GAPDH antibody. (TIF 2426 kb) [file 13041_2016_230_MOESM1_ESM.tif]

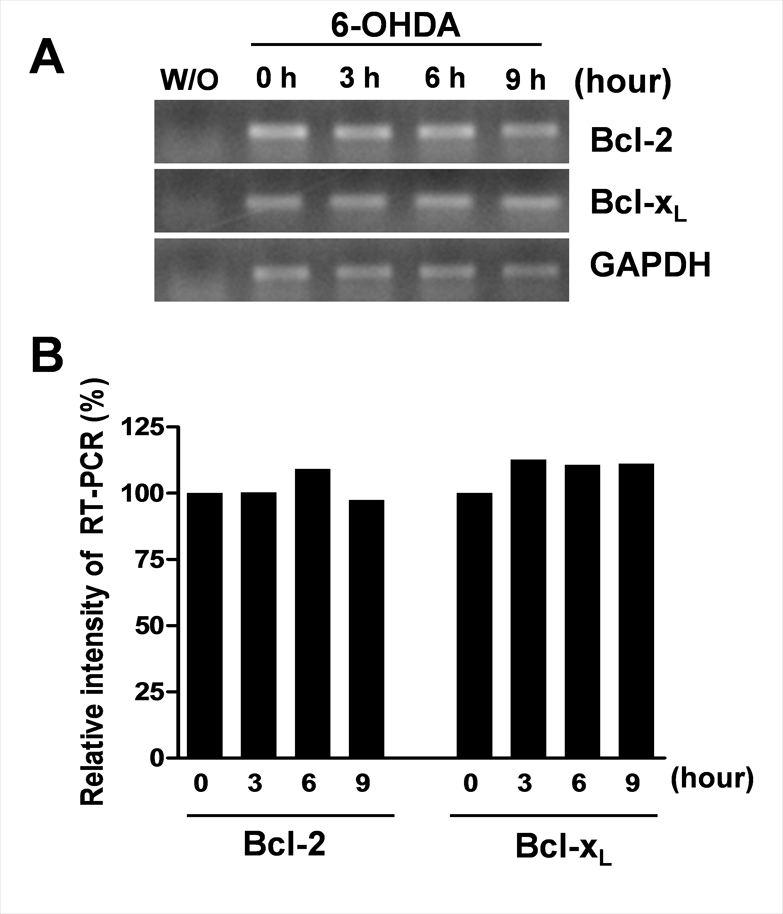

Supplement: Additional file 2: Figure S2. — Reduction of Bcl-2 level by 6-OHDA treatment is independent of transcription. a. SH-SY5Y cells were treated 50 μM 6-OHDA for 9 h and total RNA was isolated. Purified 3 μg of total RNA was used in RT-PCR analysis for Bcl-2, Bcl-XL, and GAPDH mRNA (w/o: negative control without RT reaction). b. Bar graphic representation of RT-PCR analysis. The intensity of Bcl-2 or Bcl-XL band at each time point was normalized by the intensity of GAPDH band, respectively. The data was presented a relative value to value of 0 h. (TIF 163 kb) [file 13041_2016_230_MOESM2_ESM.tif]

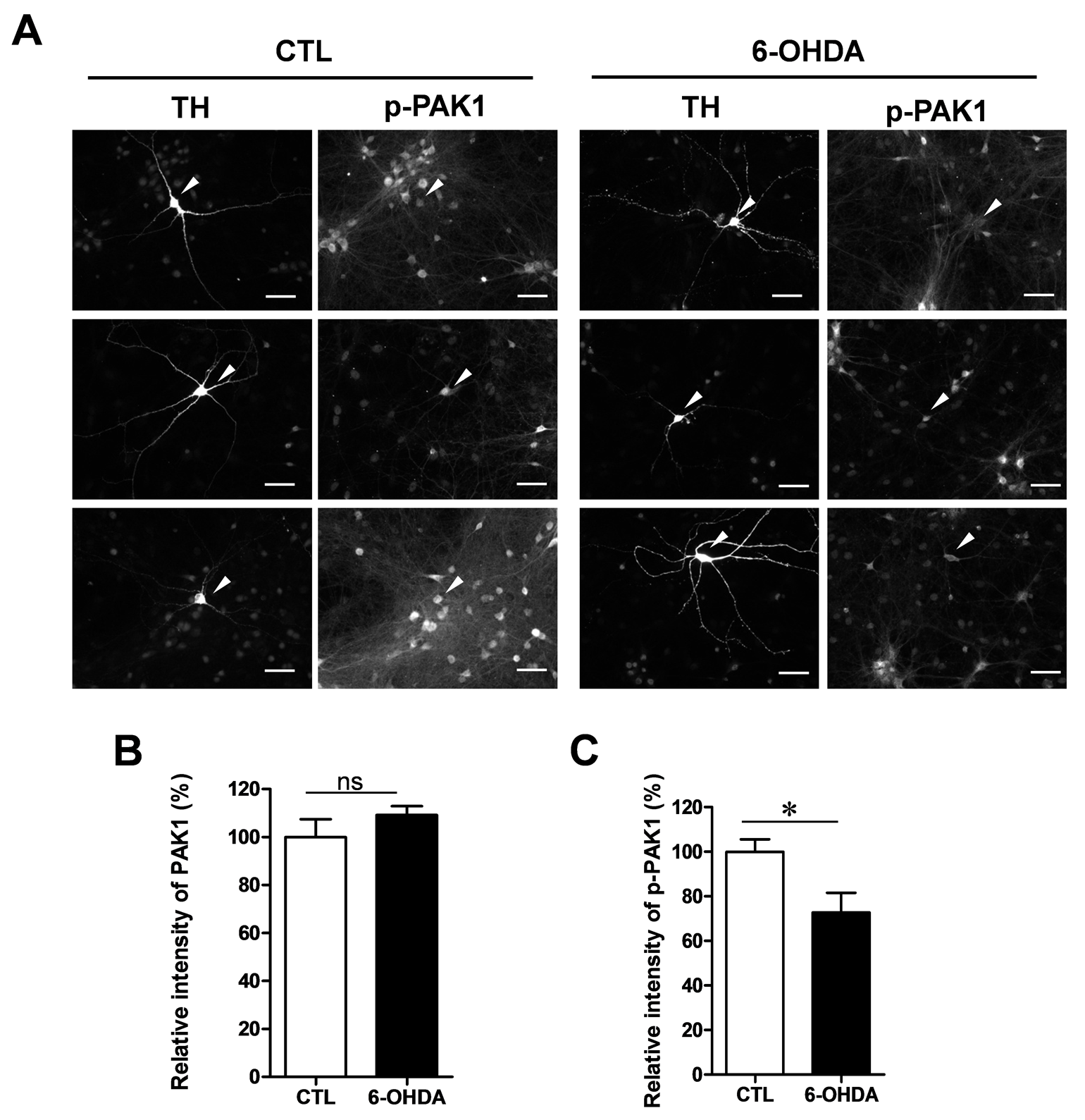

Supplement: Additional file 3: Figure S3. — 6-OHDA treatment reduces phosphorylated PAK1 level in cultured DA neurons. a. Mesencephalic DA neurons were isolated from the ventral tegumental area (VTA) and cultured for 16 day before use. Cultured DA neurons were treated with 100 μM 6-OHDA for 9 h and fixed. The cultures were immunostained with anti-monoclonal tyrosine hydroxylase antibody (TH-16, T2928, Sigma-Aldrich), anti-polyclonal PAK1 antibody (N-20, sc-882, SANTA CRUZ Biotechnology), or anti-phospho-PAK1 antibody (sc-21903-R, SANTA CRUZ Biotechnology). The cultures were subsequently immunostained with Alex Fluor® 488 anti-mouse IgG antibody (A11029, Molecular Probes) for visualization of TH staining and Cy3-conjugated anti-rabbit IgG antibody (111–165–144, Jackson ImmunoResearch Lab) for PAK1 and p-PAK1 staining. Images were acquired by fluorescent microscopy (BX-51, Olympus) and analyzed by NIH image analysis program (ImageJ ver 1.47v). Scale bar: 20 μm. b. Bar graphic representation of image analysis. 6-OHDA treatment could not change PAK1 level (CTL: 100.0 ± 7.40 %, n = 55 neurons from 6 plates; 6-OHDA: 109.2 ± 3.79 %, n = 56 neurons from 6 plates, ns: not significant) but decreased p-PAK1 level (CTL: 100.0 ± 5.56 %, n = 20 neurons from 3 plates; 6-OHDA: 72.71 ± 8.81 %, n = 27 neurons from 3 plates, Student’s t-tests: * P < 0.05) (TIF 10356 kb) [file 13041_2016_230_MOESM3_ESM.tif]

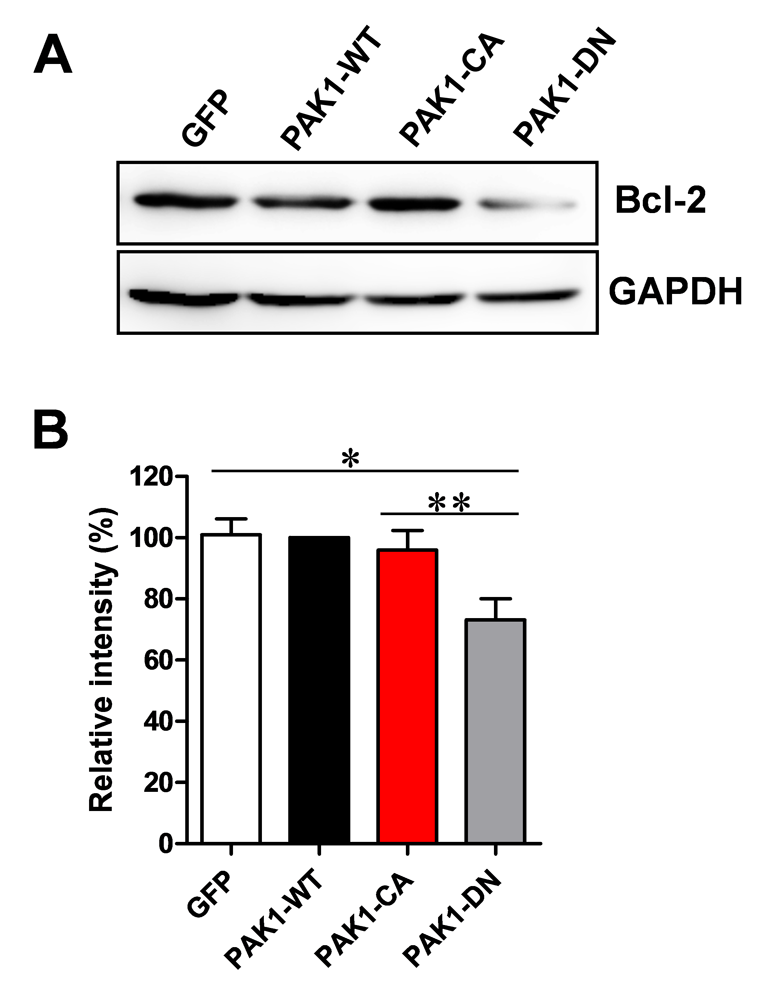

Supplement: Additional file 4: Figure S4. — PAK1-DN expression reduces the Bcl-2 level in HEK 293T cells. a. Lysates of HEK 293T cells transfected with GFP, PAK1-WT, PAK1-CA, or PAK1-DN vectors were subjected to Western blot analysis using Bcl-2 antibody. b. Quantification of Western blot analysis. Each band was normalized with the band intensity of WT (GFP: 101.0 ± 5.19 %, n = 5; WT: 100.0 %, n = 5; CA: 95.9 ± 6.46 %, n = 5; DN: 73.16 ± 6.87 %, n = 5; One-way ANOVA, F 3,19 = 5.909, ** P = 0.0065, Newman-Keuls multiple comparison test: ** P < 0.01, * P <0.5). (TIF 2613 kb) [file 13041_2016_230_MOESM4_ESM.tif]

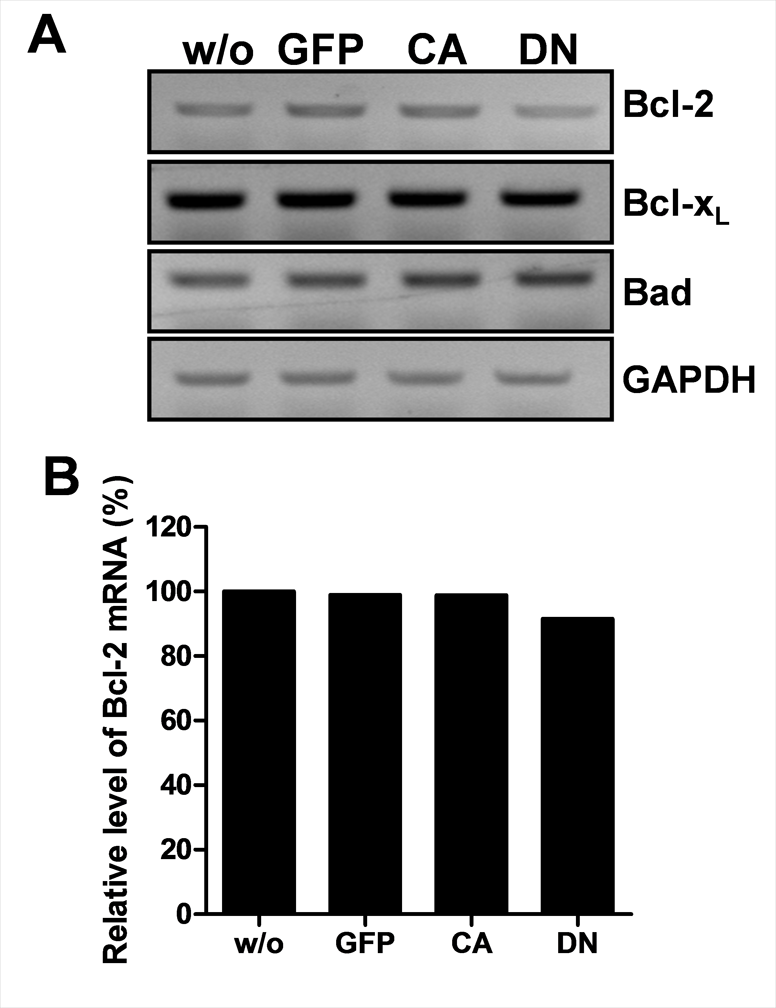

Supplement: Additional file 5: Figure S5. — Reduction of Bcl-1 by PAK-DN is independent of transcription. a. Total RNA was purified from SH-SY5Y cells expressing GFP, PAK1-CA, or PAK1-DN. And 3 μg of total RNA from each was used for RT-PCR analysis using SuperScript III (Invitrogen). The PCR primers used in RT-PCR are follows; Bcl-2 forward: 5′-agatgggaacactggtggag-3′, Bcl-2 reverse: 5′-cttccccaaaagaaatgcaa-3′, Bad forward: 5′-cctcaggcctatgcaaaaag-3′, Bad reverse: 5′-taaacctggctcgcgactta-3′, Bcl-xL forward: 5′-ggctgggatacttttgtgga-3′, Bcl-xL reverse: 5′-gggagggtagagtggatggt-3′, GAPDH forward: 5′-gagtcaacggtttggtcgt-3′, GAPDH reverse: 5′-ttgattttggagggatctcg-3′. b. Bar graphic representation of RT-PCR analysis. Each band was normalized with intensity of GAPDH. (TIF 208 kb) [file 13041_2016_230_MOESM5_ESM.tif]

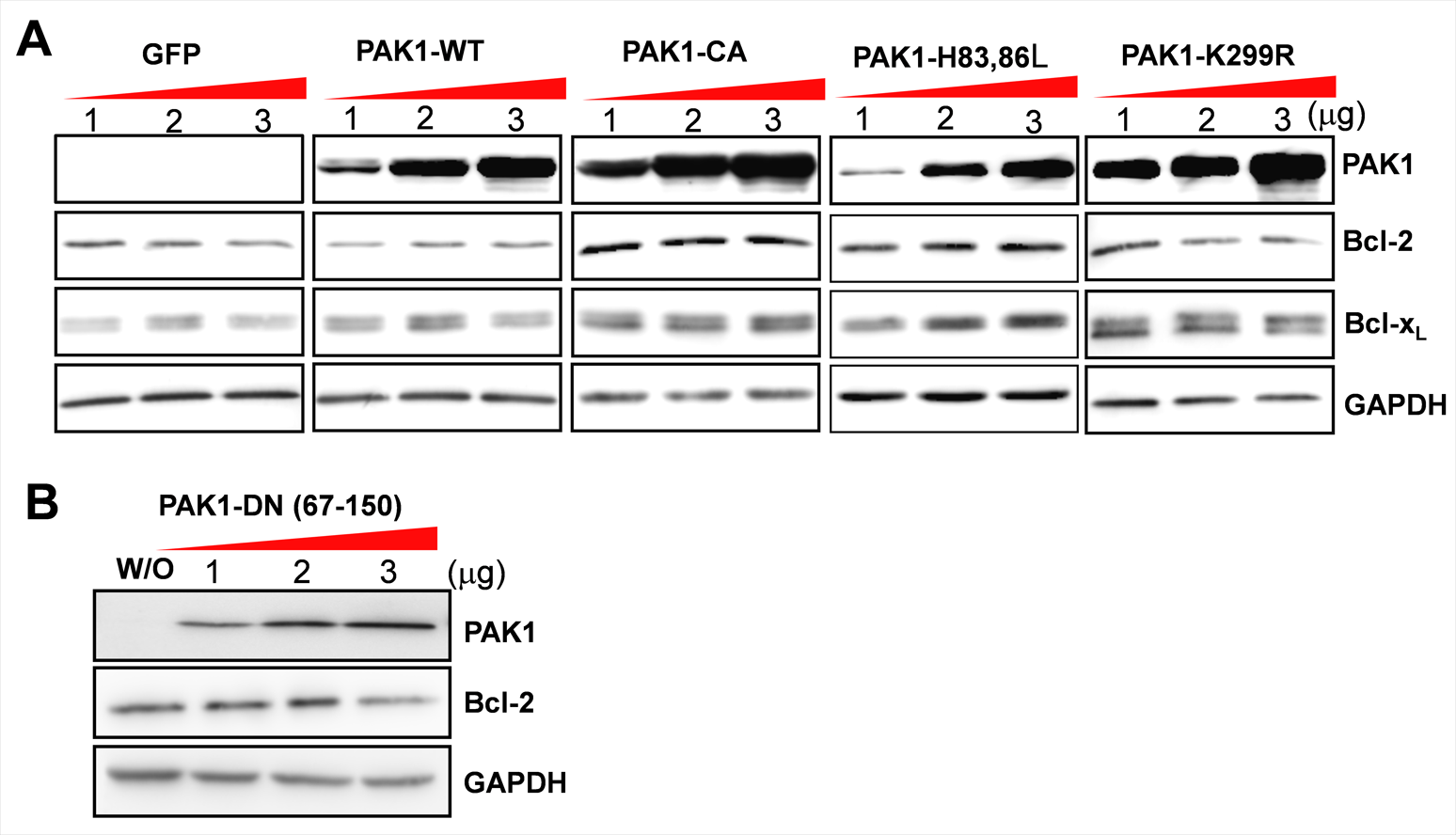

Supplement: Additional file 6: Figure S6. — Other mutants of PAK1- inactive form reduce Bcl-2 level. a. HEK cells were transfected with 1, 2, or 3 μg of GFP, PAK1-WT, PAK1-H83,86L, or PAK1-K299R vectors and incubated for 48 h. The cell lysates were subjected to Western blotting analysis using anti-Myc, Bcl-2, Bcl-xL, or GAPDH antibody. b. The autoinhibitory domain (AID) of hPAK1 (67–150 amino acids) were amplified by PCR methods (hPAk1-67-R1-S: 5′-ggaattcggaagaaagagaaagagcggc-3′, hPAK1-150-Xho-A: 5′-ccgctcgagttaagctgacttatctgtaaagc-3′) and inserted to EcoRI/XhoI site of pCMV-Myc vector (Clontech) in-frame with Myc-tag, producing PAK1-DN (67–150) [47]. HEK cells were transfected with 1, 2, or 3 μg of PAK1-DN (67–150) vector and cell lysates were analyzed by Western blotting assay. (TIF 278 kb) [file 13041_2016_230_MOESM6_ESM.tif]

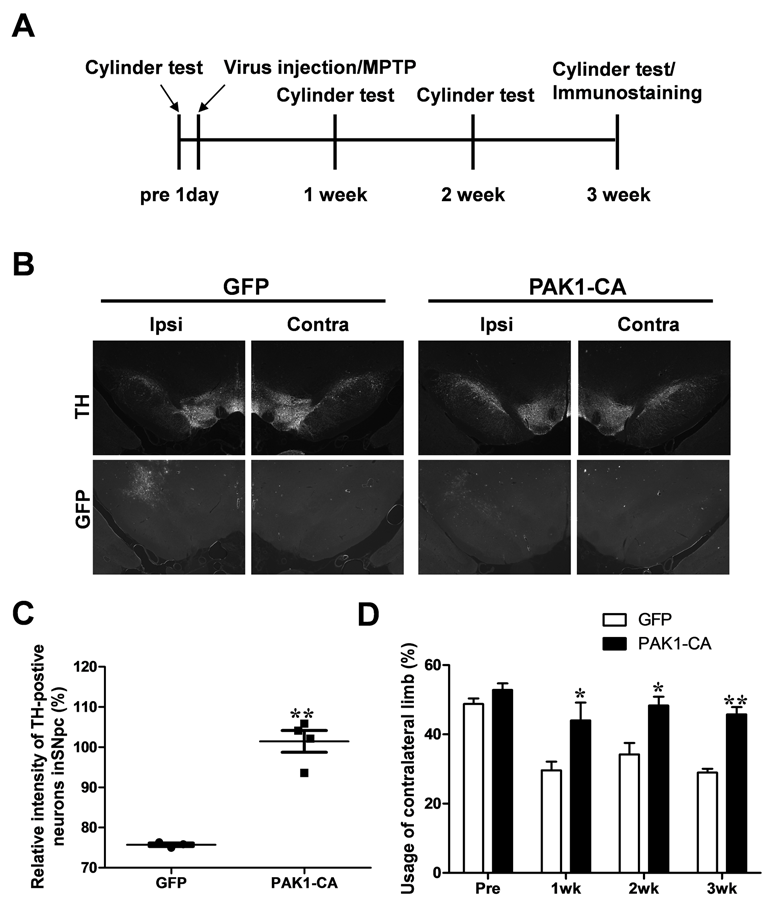

Supplement: Additional file 7: Figure S7. — PAK1-CA expression rescues behavioral defect and loss of mesencephalic dopamine neurons induced by MPTP injection. a. Experimental scheme and procedure. b. Lentiviruses encoding GFP or PAK1-CA were infused into the right SNpc at rate of 0.5 μl/min at the following coordinates: anteroposterior, −5.3 mm from the bregma; mediolateral, −2.3 mm; dorsoventral, −7.3 mm below surface of the dura. A 2 μl dose of MPTP-HCl (1 μM, 1-Methyl-4-phenyl-1,2,3,6-tetrahydropyridine hydrochloride, Sigma-Aldrich) was infused into the right striatum at a rate of 0.5 μl/min at the following coordinates: anteroposterior, 1.0 mm from the bregma; mediolateral, −3.0 mm; dorsoventral, −5.0 mm below surface of the dura [48]. After 3 weeks, the midbrain tissue sections were immunostained with anti-Tyrosine Hyroxylase antibody and visualized by immunostaining using Cy3-conjugated goat anti-rabbit IgG antibody. c. The loss of DA neurons in the SNpc induced by MPTP injection was blocked by PAK1-CA expression, but not by GFP expression (GFP: 75.7 ± 0.41 %, n = 3, compared to the ipsilateral side; PAK1-CA: 101.4 ± 2.71 %, n = 4, compare to ipsilateral side, Student’s t-tests, **P < 0.01). d. PAK1-CA expression significantly improved usage of the contralateral limb in a hemiparkinsonian animal model (GFP: PAK1-CA, pretest, 48.8 ± 1.59, n = 3: 52.8 ± 1.92, n = 4; 1 week, 29.6 ± 2.50, n = 3: 44.03 ± 5.18, n = 4; 2 weeks, 34.2 ± 3.30, n = 3: 48.30 ± 2.58, n = 4; 3 weeks, 28.97 ± 1.09, n = 3: 45.75 ± 2.15, n = 4; Two-way ANOVA, F 3, 20 = 1.746, P = 0.19, 1, Bonferroni multiple comparison tests, *P < 0.05, **P < 0.01). (TIF 2786 kb) [file 13041_2016_230_MOESM7_ESM.tif]
